# Predictors and burden of injury mortality in the Thai cohort study 2005–2015

**DOI:** 10.1186/s12889-020-09803-1

**Published:** 2020-11-16

**Authors:** C. T. Lowe, M. Kelly, S. Seubsman, A. Sleigh

**Affiliations:** 1 Department of Global Health, Research School of Population Health, Australian National University, 339/4 Hutton St, Acton, ACT 2601 Australia; 2 School of Human Ecology, Sukhothai Thammathirat Open University, Nonthaburi, Thailand

**Keywords:** Mortality, Cohort study, Epidemiology, Risks/ determinants, Low-middle income country

## Abstract

**Background:**

Thailand is a high injury burden setting. In 2015 it had the world’s second highest rate of road traffic fatalities. In order to develop strategies to reduce this burden an accurate understanding of the development of injury risk over the life course is essential.

**Methods:**

A national cohort of adult Thais was recruited in 2005 (*n* = 87,151). Participants completed a health questionnaire covering geodemographic, behavioural, health and injury data. Citizen ID numbers were matched with death registration records, identifying deaths from any injury. Adjusted logistic regression models were used to measure associations between baseline exposures and injury deaths between 2005 and 2015.

**Results:**

Injury mortality comprised 363 individuals, the majority (36%) from traffic injuries. Predictors of all-injury mortality were being male (AOR 3.55, 95% CI 2.57–4.89), Southern Thai (AOR 1.52, 95% CI 1.07–2.16), smoking (AOR 1.55, 95% CI 1.16–2.17), depression (AOR 1.78, 95% CI 1.07–2.96), previous injury (AOR 1.37, 95% CI 1.03–1.81) and drink driving history (AOR 1.37, 95%CI 1.02–1.85). Age and region of residence were stronger predictors for men, while anxiety/depression was a stronger predictor for women. Among males in the far south, assault caused the largest proportion of injury mortality, elsewhere traffic injury was most common.

**Conclusions:**

This study identifies that a history of drink driving, but not regular alcohol consumption, increased injury risk. The associations between smoking and depression, and injury mortality also need further consideration.

## Background

Injury causes significant morbidity and mortality globally. Approximately one-tenth of global deaths are injury-related [1], and road traffic injuries proceeded by suicide were the fourth and sixth leading causes of death globally for 15–49 year olds in 2017 [2]. Injury deaths may also include poisonings, falls, fire-related deaths, drowning, intentional self-harm, assault as well as other un-intentional injuries. The distribution of injuries however is not equal globally; approximately 90% of all injury deaths occur in low- and middle-income countries [1, 3].

In recent decades in high income countries the implementation of interventions such as speed limit enforcement, drink-driving testing and smoke detectors has led to a reduction in the burden of injuries [1]. Impeding on the ability for the governments of low and middle income countries to design effective injury-prevention strategies is the lack of data, evidence [3] and resources [4]. Often the processes of recording data for injury deaths in these countries are not complete, police reports, hospital based data and national vital registration statistics regularly are not integrated and have large discrepancies in reporting the burden of injury deaths and lead to an under-represented figure. As such the burden of injuries, the relative burden of various injury classifications, and the distribution of injury burden amongst population sub-groups are still poorly understood in low- and middle-income countries. Furthermore, injuries are a difficult cause of death to monitor because of the complexities associated with defining the specific cause of injury death. One problem often contributing to injury death rates being underestimated is when a death occurs due to an injury, however only the immediate cause of the injury is recorded as the cause of death (bleeding, fractures etc.) and the underlying cause of an injury (traffic accident, fall, assault etc.) is lost in health data.

Thailand, a south-east Asian country, has been reported as a major contributor of global injury mortality. Thailand has been reported to have the highest rate of road-traffic accidents globally (36.2 per 100,000 population in 2015) [5]. Increasing mortality due to road-traffic accidents in such low- and middle-income countries has been attributed to rapid economic development which has outpaced road infrastructure development and safety provisions [6]. As an ageing population, fall-deaths among the elderly also contribute to injury mortality in Thailand [7]. Furthermore, many injury deaths are initially recorded with undetermined intent [8] and many with no known underlying injury. However, studies using verbal autopsies to investigate details surrounding injury deaths have resulted in more specific cause of death data in Thailand. In particular, deaths due to suicide, assault and drowning have been more accurately estimated [9]. Other studies have resulted in an increase of mortality cases being assigned to road-traffic injury through examining details of these non-specific cause fatalities [10]. It has also been found in Thailand that physicians who were young and inexperienced and older physicians frequently mis-classified the underlying cause of deaths [8]. Techniques for re-distributing the classification of non-specific injury deaths into specific underlying causes have been developed in order to improve the validity of injury-burden calculations, one such technique using demographics and characteristics of groups with known-cause injury deaths and comparing these characteristics with unknown-cause injury deaths for allocation [11].

After an accurate calculation of the burden of injury in Thailand, understanding risk factors and socio/geodemographic groups at high risk to injury mortality assists in developing effective injury prevention strategies. Alcohol use, [12] depression, [13, 14] and smoking incidence [15] have consistently been reported as risk factors for injury mortality. Young males are consistently reported as the demographic with highest risk of injury mortality in Thailand [16]. Alcohol is a major risk factor for injury mortality for all demographics of any population, and this risk is particularly dangerous for road-traffic accidents as it negatively effects drivers’ judgement and reaction time [17].

As such, we aim to investigate long-term risk factors for injury mortality in Thailand and the distribution of various injury types. In doing so, we can identify specific demographics and risk factors towards injury mortality later in an individual’s life. Thus, interventions towards injury mortality in Thailand can be developed in order to reduce the burden of injury mortality and particularly pre-mature death due to injury. Our research here is an extension to our prior work on injury deaths in the Thai Cohort Study (TCS), a study of 87,134 students enrolled at Sukhothai Thammathirat Open University (STOU). We have previously investigated injury mortality in our cohort for deaths occurring between 2005 to 2010 [4]. Here, we extend our research to cover deaths from 2005 to 2015 to investigate the burden of and risk factors for injury mortality in the cohort.

## Methods

### Study population and data collection

This analysis is part of the Thai Cohort Study (TCS); a longitudinal epidemiological investigation of health risks and outcomes in Thailand. A cohort of 87,134 distance-learning students residing all over Thailand enrolled at Sukhothai Thammathirat Open University (STOU) who completed the baseline mail-out 20-page health questionnaire in 2005 forms the study population for this analysis. This questionnaire was mailed to approximately 200,000 STOU students and by February 2006 a total of 87,134 had returned completed questionnaires and consent forms.

At baseline the cohort members were aged between 15 and 87 years and were similar to the general Thai population in terms of median age (29 years), sex distribution (54% female compare to 51% in general population), median income (USD2550 vs USD 2742 in the general population), religion (94% Buddhist in both populations) and geographic distribution across regions of Thailand. The cohort was however 51% urban versus 31% in the general population, had a younger overall age structure with 52% being aged between 20 and 29 years, and had a higher average level of education (96% had finished high school, versus 21% in the national population in 2005) [18].

Questions were split into multiple categories, including ‘You and your home’, ‘Income and work’, ‘Your health, injuries and health service use’, ‘Social networks and well-being’, ‘Food and physical activity’, ‘Tobacco, alcohol and transport’ and ‘Your family’.

### Mortality data

Our analysis involved measuring associations between demographic and health-risk factors from baseline in 2005 and mortality between 2005 and 2015. Mortality data was obtained using the Thai citizen ID number provided by all participants at baseline. At various time points, the citizen ID numbers of participants in the cohort were matched with the Thai Ministry of Interior to obtain lists of participants in the cohort who had died by the time point. This list was then sent to the Thai Ministry of Public Health, where the WHO ICD code (International Classification of Disease code) was added to provide information on the cause of death. A previous paper has been published on injury mortality deaths up to 2010 (total 204) within the cohort (Yiengprugsawan et al., 2014). We now have data on deaths occurring in the cohort up to 2016. We observed 1232 deaths up to December 2015, 363 (29.5%) of which were injury deaths. For a death to be classified as an injury death in our analysis, it had to have an ICD-10 code for cause of death included in and between chapters V to Y. These deaths were further grouped into a condensed list, consisting of *Transport accidents, Assault, Intentional self-harm, Drowning, Falls, Exposure to smoke, fire and flames, and All other external causes*.

### Exposures and confounders

Exposures and confounders of interest to our analysis included geo-demographic and health-risk variables. Age was stratified and calculated as the age at midpoint (2010) as this best represents the distribution of the ageing cohort over the 10-year period. It was not practical to measure age as a continuous variable because the association with age and the log odds of injury mortality was not linearly related. Other geo-demographic variables included sex, current residence (urban/rural as well as region) at baseline, highest education level excluding participation at STOU, and personal monthly income.

Health-risk variables we were interested in included alcohol drinking habits (never, occasional drinkers and regular drinkers), smoking (never smoked, have quit smoking before baseline, current smokers at baseline), drink-driving (question posed as having drunk 3 or more glasses of alcohol and then driven a motor vehicle in the 12 months prior to answering the survey at baseline), a doctor diagnosis of anxiety/depression, as well as reporting having experienced non-fatal injuries in the 12 months prior to baseline.

### Data processing and statistical analysis

Analysis of variables was performed in SPSS software (SPSS) [19].

We first quantified the distribution of the condensed causes of injury mortality in the cohort (*Transport accidents*, *Assaults*, *Intentional self-harm*, *Accidental drowning*, *Falls*, Smoke, fire and flames and *All other and external causes*).

Next, we proceed to analysis for risk factors and geodemographic groups at higher risk for injury mortality in the cohort. First, the distribution of mortality by baseline social and demographic attributes was calculated, as well as for all exposures and confounders of interest. The variables chosen to include in analysis were determined by prior literature review, as well as having an initial significant association with injury death. Crude odds ratios and corresponding *p*-values were calculated. Predictors of injury mortality that had significant associations (*p* < 0.05) with injury mortality in this initial analysis were incorporated into a binary logistic regression for injury mortality in the cohort where we present a fully mutually-adjusted odds ratio. A cox regression was calculated with the same variables as those entered in the final binary logistic regression. Adjusted hazard ratios were close to one, indicating no association between survival time and injury mortality. As such, no survival analysis is included in this report. We then performed a multivariate logistic regression for injury/non-injury mortality, both for the entire cohort and stratified by sex, calculating crude and adjusted odds ratios. Reference category for the outcome variable is being alive by endpoint.

### Ethics approval

Ethics approval was obtained from Sukhothai Thammathirat Open University Research and Development Institute (protocol 0522/10) and the Australian National University Human Research Ethics Committee (protocols 2,004,344 and 2009/570). Informed written consent was obtained from all participants. In the baseline recruitment process participants were requested to provide their Citizen Identification Number and informed that the study would use this to identify vital events among the participants. The number was supplied voluntarily. These confidential ID numbers were safeguarded and stored at STOU in a secure office on the main campus with 24 h guards on patrol. The working files of these data were de-identified and no individual information will be released or displayed in any format.

## Results

In Table 1 we present the distribution of injury causes in the cohort. From 2005 to 2015, there were 363 deaths in the cohort which accounted for approximately a quarter of all deaths. Of all injury deaths, about one third were due to all other external causes (predominantly unknown-cause injuries) and a third were transport accidents. Following transport accidents was assault making up about 11.6% of all injuries, intentional self-harm making up about 7%, accidental drowning (5%) and a very small number of falls and smoke/fire/flames deaths.

**Table 1:** Distribution of injury mortality by underlying injury cause, Thai Cohort Study 2005–2015

Cause of injury death	n (%)
Transport accident	131 (36.1)
Assault	42 (11.6)
Intentional self-harm	26 (7.2)
Accidental drowning	18 (5.0)
Falls	7 (1.9)
Exposure to smoke, fire and flames	2 (0.6)
All other external causes	137 (37.7)
**Total**	363 (100.0)

In Table 2, we first present the distribution of injury deaths in the cohort by all risk factors and present the crude odds ratio, and then proceed with the findings from a fully-adjusted multivariate logistic regression model for injury/non-injury mortality. Risk factors for injury mortality initially included age above 60 years, male sex, rural and southern Thailand residence, historical smoking and alcohol consumption, anxiety/depression, injury history and drink-driving history. University and diploma/certificate education were a protector for injury mortality. When all variables were incorporated into a fully mutually-adjusted model, male sex (AOR 3.39, 95% CI 2.47–4.66), southern regency (AOR 1.52, 95% CI 1.06–2.16), baseline smoker status (AOR 1.52, 95% CI 1.09–2.11), anxiety/depression (AOR 1.87, 95% CI 1.07–2.96), injury history (AOR 1.37, 95% CI 1.03–1.81) and drink-driving (AOR 1.37, 95% CI 1.14–3.07) remained significant predictors of injury mortality. Alcohol consumption was confounded by male sex and education was confounded by age (data not shown). Overall, male sex had the strongest association with injury mortality, and was more strongly associated with injury mortality (AOR 3.39) than non-injury mortality (AOR 1.43). Smoking, anxiety/depression and prior injuries also had an association with non-injury mortality in the fully adjusted model, however drink-driving was protective to non-injury mortality.

**Table 2:** Multivariate predictors for injury and non-injury mortality, Thai Cohort Study

	Univariate^b^				Multivariate^b^	
	Injury mortality		Non-injury mortality		Injury mortality	Non-injury mortality
	n (%)	OR (95% CI)	n (%)	OR (95% CI)	AOR (95% CI)	AOR (95% CI)
**Age group**						
15–29	104 (0.4)	–	100 (0.4)	–	–	–
30–44	196 (0.4)	0.91 (0.7–1.2)	403 (0.8)	**1.95 (1.6–2.4)**	0.77 (0.59–1.01)	**1.73 (1.35–2.20)**
45–59	53 (0.4)	1.02 (0.7–1.4)	285 (2.3)	**5.71 (4.5–7.1)**	0.79 (0.53–1.18)	**4.57 (3.51–5.94)**
60+	10 (1.3)	**3.28 (1.7–6.3)**	81 (10.2)	**27.59 (20.6–37.8)**	1.95 (0.77–4.91)	**18.36 (12.4–27.0)**
**Sex**						
Male	280 (0.7)	**4.14 (3.2–5.3)**	575 (1.5)	**2.40 (2.1–2.8)**	**3.39 (2.47–4.66)**	**1.43 (1.16–1.76)**
Female	83 (0.2)	–	294 (0.6)	–	–	–
**Highest education**						
Junior High School	20 (0.7)	–	73 (2.4)	–	–	–
High School	194 (0.5)	0.73 (0.5–1.2)	388 (1)	**0.40 (0.3–0.5)**	1.01 (0.54–1.89)	0.82 (0.58–1.15)
Dip./Cert.^a^	96 (0.4)	0.61 (0.4–1.0)	213 (0.9)	**0.37 (0.3–0.5)**	1.13 (0.59–2.14)	0.90 (0.63–1.28)
University	53 (0.3)	0.38 (0.2–0.6)	192 (0.9)	**0.37 (0.3–0.5)**	0.70 (0.36–1.37)	**0.67 (0.47–0.95)**
**Home location**						
Urban	159 (0.4)	–	422 (0.9)	–	–	–
Rural	201 (0.5)	**1.36 (1.1–1.7)**	438 (1.1)	1.12 (0.9–1.3)	1.18 (0.92–1.52)	**1.19 (1.00–1.41)**
**Region**						
Central/East	94 (0.4)	–	231 (0.9)	–	–	–
Bangkok	43 (0.3)	0.82 (0.6–1.2)	156 (1.1)	1.21 (0.9–1.5)	1.01 (0.68–1.52)	1.17 (0.91–1.50)
North	70 (0.4)	1.26 (0.9–1.7)	165 (1)	1.20 (0.9–1.5)	1.17 (0.83–1.66)	1.09 (0.86–1.39)
Northeast	85 (0.5)	1.33 (0.9–1.8)	210 (1.2)	**1.34 (1.1–1.6)**	1.02 (0.72–1.43)	**1.25 (1.00–1.56)**
South	69 (0.6)	**1.73 (1.3–2.4)**	100 (0.9)	1.02 (0.8–1.3)	**1.52 (1.06–2.16)**	1.12 (0.86–1.46)
**Smoking**						
Currently	94 (1.1)	**3.76 (2.9–4.8)**	179 (2)	**2.99 (2.5–3.6)**	**1.52 (1.09–2.11)**	**1.84 (1.43–2.36)**
Quit	79 (0.5)	**1.87 (1.4–2.4)**	233 (1.6)	**2.30 (1.9–2.7)**	0.87 (0.62–1.23)	1.22 (0.97–1.55)
Never	178 (0.3)	–	427 (0.7)	–	–	–
**Alcohol consumption**						
Regularly	33 (0.8)	**2.87 (1.8–4.4)**	86 (2.1)	**2.80 (2.2–3.6)**	0.83 (0.48–1.41)	1.34 (0.96–1.88)
Occasionally	212 (0.4)	**1.47 (1.1–1.9)**	448 (0.9)	1.16 (0.9–1.4)	0.79 (0.56–1.11)	0.88 (0.71–1.08)
Never	64 (0.3)	–	171 (0.8)	–	–	–
**Anxiety/Depression**						
Yes	22 (0.7)	**1.83 (1.2–2.8)**	43 (1.4)	**1.48 (1.1–2.0)**	**1.87 (1.14–3.07)**	**1.53 (1.07–2.19)**
No	341 (0.4)	–	823 (1)	–	–	–
**Prior injuries**						
One or more	84 (0.6)	**1.54 (1.2–1.9)**	188 (1.3)	**1.47 (1.2–1.7)**	**1.34 (1.02–1.77)**	**1.37 (1.13–1.66)**
None	265 (0.4)	–	624 (0.9)	–	–	–
**Drink driving**						
Yes	159 (0.7)	**2.30 (1.9–2.8)**	256 (1.2)	**1.24 (1.1–1.4)**	**1.38 (1.03–1.85)**	**0.78 (0.64–0.96)**
No	200 (0.3)	–	598 (0.9)	–	–	–

^a^Diploma/Certificate. ^b^Reference category is being alive by endpoint

Odds ratios in bold typeface are statistically significant at *p* < 0.05

In Table 3, we present the results from a multivariate logistic regression for injury/non-injury mortality among females with the same variables as those presented in Table 2 excluding urban/rural residence. Southern region residency and smoking were not significant predictors among females, however those who reported occasionally drinking alcohol had half the odds of injury mortality compared to females who have never drunk alcohol (AOR 0.52, 95% CI 0.31–0.86). Anxiety/Depression was also a stronger predictor in females (AOR 2.98, 95% CI 1.35–6.58). Prior injuries was no longer associated with injury mortality, but was associated with non-injury mortality (AOR 1.65, 95% CI 1.22–2.23).

**Table 3:** Multivariate predictors for injury and non-injury mortality among females, Thai Cohort Study

	Univariate^b^				Multivariate^b^	
	Injury mortality		Non-injury mortality		Injury mortality	Non-injury mortality
	n (%)	OR (95% CI)	n (%)	OR (95% CI)	AOR (95% CI)	AOR (95% CI)
**Age**						
15–29	29 (0.2)	–	53 (0.3)	–	–	–
30–44	40 (0.2)	0.83 (0.5–1.3)	165 (0.6)	**1.88 (1.4–2.6)**	1.08 (0.64–1.82)	1.89 (1.36–2.64)
45–59	14 (0.3)	1.59 (0.8–3.0)	73 (1.5)	**4.54 (3.2–6.5)**	1.94 (0.95–3.93)	4.75 (3.23–6.99)
60+	0 (0)	0	3 (1.8)	**5.36 (1.7–17.3)**	–	7.07 (2.15–23.2)
**Education**						
Junior High School	1 (0.1)	–	7 (0.7)	–	–	–
High School	36 (0.2)	1.96 (0.3–14.3)	136 (0.7)	1.06 (0.5–2.3)	1.54 (0.20–11.4)	1.20 (0.55–2.62)
Dip./Cert.^a^	32 (0.2)	2.34 (0.3–17.2)	86 (0.6)	0.90 (0.4–1.9)	2.16 (0.29–16.0)	1.07 (0.48–2.36)
University	14 (0.1)	1.22 (0.2–9.3)	64 (0.5)	0.79 (0.4–1.7)	1.03 (0.13–8.00)	0.72 (0.32–1.62)
**Region**						
Central/East	24 (0.2)	–	84 (0.6)	–	–	–
Bangkok	10 (0.1)	0.70 (0.3–1.5)	72 (0.8)	**1.43 (1.0–2.0)**	0.71 (0.32–1.55)	1.21 (0.85–1.72)
North	17 (0.2)	1.33 (0.7–2.5)	49 (0.6)	1.10 (0.8–1.6)	1.36 (0.69–2.66)	1.12 (0.76–1.66)
Northeast	19 (0.2)	1.42 (0.8–2.6)	46 (0.5)	0.98 (0.7–1.4)	1.32 (0.69–2.54)	1.01 (0.68–1.50)
South	12 (0.2)	1.20 (0.6–2.4)	42 (0.7)	1.20 (0.8–1.7)	1.10 (0.53–2.28)	1.40 (0.94–2.07)
**Smoking**						
Currently	2 (0.4)	2.43 (0.6–9.9)	9 (1.9)	**3.21 (1.6–6.3)**	1.42 (0.17–11.5)	**2.68 (1.24–5.79)**
Quit	4 (0.2)	1.06 (0.4–2.9)	15 (0.7)	1.17 (0.7–2.0)	1.15 (0.34–3.83)	0.91 (0.47–1.75)
Never	76 (0.2)	–	259 (0.6)	–	–	–
**Alcohol**						
Regularly	1 (0.3)	1.45 (0.2–10.5)	5 (1.6)	**2.71 (1.1–6.7)**	1.08 (0.12–9.27)	2.15 (0.79–5.82)
Occasionally	33 (0.1)	**0.59 (0.4–0.9)**	147 (0.6)	0.98 (0.8–1.3)	**0.52 (0.31–0.86)**	1.05 (0.80–1.37)
Never	42 (0.2)	–	112 (0.6)	–	–	–
**Anxiety/depression**						
Yes	7 (0.4)	**2.49 (1.1–5.4)**	13 (0.8)	1.25 (0.7–2.2)	**2.98 (1.35–6.58)**	1.20 (0.65–2.21)
No	76 (0.2)	–	281 (0.6)	–	–	–
**Prior injuries**						
One or more	17 (0.2)	1.42 (0.8–2.4)	65 (0.9)	**1.62 (1.2–2.1)**	1.43 (0.80–2.55)	**1.65 (1.22–2.23)**
None	65 (0.2)	–	217 (0.6)	–	–	–
**Drink-driving**						
Yes	8 (0.2)	1.15 (0.6–2.4)	21 (0.5)	0.85 (0.5–1.3)	1.25 (0.52–3.01)	0.73 (0.43–1.23)
No	75 (0.2)	–	267 (0.6)	–	–	–

^a^Diploma/Certificate. ^b^Reference category is being alive by endpoint

Odds ratios in bold typeface are statistically significant at *p* < 0.05

Results from the multivariate logistic regression for injury/non-injury mortality among males is presented in Table 4. Whilst adjusted odds of non-injury mortality increased with age, middle-age was protective to injury mortality (AOR 0.53, 95% CI 0.33–0.85 for injury mortality among 45–59 year olds compared to 15–29 year olds). Residing in southern Thailand remained significantly associated with injury mortality (AOR 1.79, 95% CI 1.20–1.68), as well as currently smoking at baseline (AOR 1.51, 95% CI 1.08–2.12). Anxiety/Depression was no longer significantly associated with injury mortality in males, but was significantly associated with non-injury mortality (AOR 1.71, 95% CI 1.09–2.66). Drink-driving was significantly associated with injury mortality (AOR 1.37, 95% CI 1.00–1.87), and protected against non-injury mortality, however this was not statistically significant (AOR 0.81, 95% CI 0.64–1.02).

**Table 4:** Multivariate predictors for injury and non-injury mortality among males, Thai Cohort Study

	Univariate^b^				Multivariate^b^	
	Injury mortality		Non-injury mortality		Injury mortality	Non-injury mortality
	n (%)	OR (95% CI)	n (%)	OR (95% CI)	AOR (95% CI)	AOR (95% CI)
**Age**						
15–29	75 (0.9)	–	47 (0.6)	–	–	–
30–44	157 (0.7)	**0.72 (0.5–0.9)**	238 (1)	**1.75 (1.3–2.4)**	**0.64 (0.46–0.87)**	**1.57 (1.11–2.23)**
45–59	38 (0.5)	**0.57 (0.4–0.8)**	211 (2.9)	**5.03 (3.6–6.9)**	**0.53 (0.33–0.85)**	**4.29 (2.97–6.18)**
60+	10 (1.6)	**1.99 (1.0–3.9)**	79 (12.7)	**25.08 (17.3–36.4)**	1.78 (0.70–4.53)	**18.7 (11.7–29.7)**
**Education**						
Junior High School	19 (1)	–	66 (3.4)	–	–	–
High School	158 (0.8)	0.81 (0.5–1.3)	252 (1.3)	**0.37 (0.3–0.5)**	0.94 (0.48–1.82)	0.73 (0.50–1.06)
Dip./Cert.^a^	64 (0.7)	0.73 (0.4–1.2)	127 (1.4)	**0.42 (0.3–0.6)**	0.94 (0.47–1.88)	0.91 (0.61–1.36)
University	39 (0.4)	**0.45 (0.3–0.8)**	128 (1.5)	**0.43 (0.3–0.6)**	0.65 (0.32–1.34)	0.71 (0.47–1.06)
**Region**						
Central/East	70 (0.6)	–	147 (1.3)	–	–	–
Bangkok	33 (0.6)	0.93 (0.6–1.4)	84 (1.5)	1.13 (0.9–1.5)	1.07 (0.67–1.70)	1.01 (0.73–1.40)
North	53 (0.7)	1.12 (0.8–1.6)	116 (1.5)	1.16 (0.9–1.5)	1.17 (0.78–1.74)	1.12 (0.83–1.52)
Northeast	66 (0.7)	1.12 (0.8–1.6)	164 (1.7)	**1.32 (1.1–1.7)**	1.02 (0.69–1.51)	**1.43 (1.09–1.88)**
South	57 (1.2)	**1.86 (1.3–2.6)**	58 (1.2)	0.90 (0.7–1.2)	**1.79 (1.20–2.68)**	0.97 (0.68–1.39)
**Smoking**						
Currently	92 (1.1)	**1.95 (1.5–2.6)**	170 (2.1)	**2.19 (1.8–2.7)**	**1.51 (1.08–2.12)**	**1.92 (1.47–2.51)**
Quit	75 (0.6)	1.04 (0.8–1.4)	218 (1.7)	**1.84 (1.5–2.3)**	0.85 (0.60–1.22)	**1.29 (1.00–1.67)**
Never	102 (0.6)	–	168 (1)	–	–	–
**Alcohol**						
Regularly	32 (0.8)	1.58 (0.9–2.7)	81 (2.1)	**1.49 (1.1–2.1)**	1.17 (0.61–2.23)	0.97 (0.63–1.46)
Occasionally	179 (0.7)	1.26 (0.8–2.0)	301 (1.1)	0.79 (0.6–1.0)	1.10 (0.67–1.81)	**0.63 (0.45–0.87)**
Never	22 (0.5)	–	59 (1.4)	–	–	–
**Anxiety/depression**						
Yes	15 (1.2)	**1.71 (1.0–2.9)**	30 (2.4)	**1.67 (1.1–2.4)**	1.49 (0.78–2.83)	**1.71 (1.09–2.66)**
No	265 (0.7)	–	545 (1.4)	–	–	–
**Prior injuries**						
One or more	67 (0.9)	**1.44 (1.1–1.9)**	123 (1.7)	**1.30 (1.1–1.6)**	1.28 (0.93–1.75)	1.20 (0.94–1.54)
None	200 (0.7)	–	407 (1.3)	–	–	–
**Drink-driving**						
Yes	151 (0.8)	**1.37 (1.1–1.7)**	235 (1.3)	**0.81 (0.7–0.9)**	**1.37 (1.00–1.87)**	0.81 (0.64–1.02)
No	125 (0.6)	–	331 (1.6)	–	–	–

^a^Diploma/Certificate. ^b^Reference category is being alive by endpoint

Odds ratios in bold typeface are statistically significant at p < 0.05

## Discussion

Our analysis of injuries in the Thai Cohort Study found 363 injury deaths occurring between 2005 and 2015, 78% more deaths than those included in our previous report for injury deaths between 2005 – March 2010.

After mutually adjusting for all covariates, risk factors with a statistically significant association with injury mortality were being male, drink driving, current smoker status at baseline, a diagnosis of anxiety/depression, a history of injuries and residing in the south (Table 2). Being aged 60+ had strong adjusted odds of injury mortality (AOR 1.95) compared to those aged 15–29, however this association was not statistically significant. When the multivariate analysis was stratified by sex, among males, those aged between 30 and 59 had significantly smaller odds of injury mortality compared to younger participants. Smoking at baseline, southern border residency and drink driving remained significant predictors of injury mortality, whilst anxiety/depresson and prior injury were positive predictors but not statistically significant, likely due to smaller numbers after stratifying by sex (Table 4). Among females, drink-driving, smoking at baseline and prior injuries were positive but insignificant predictors of injury mortality, likely due to very few female injury deaths in the cohort. Anxiety/Depression was a bigger predictor of injury mortality in females (AOR 2.98, 95% CI 1.35–6.58) than in males. Females whose highest education level was a diploma/certificate had twice the odds of injury mortality compared to university educated females, and occasional alcohol drinkers had half the odds of injury mortality compared to females who reported never having drunk alcohol.

Interestingly, drink-driving was a significant predictor of injury mortality in the cohort, but historical alcohol consumption was not. Alcohol is widely recognised as a risk factor for injury mortality [20]. Most studies that evaluate this association measure alcohol consumption around the time of an injury, such as 6 h prior [21] or the blood-alcohol concentration immediately after an injury [22] Thus, the association between drink-driving and injury mortality in the cohort is supported by a plethora of studies, all pointing to the immediate detrimental effects of alcohol on judgement and motor ability. Historical alcohol consumption therefore may not be a predictor for injury mortality if regular drinkers in the cohort did not engage in alcohol consumption prior to a potential injury hazard, such as before driving. However other studies have found historical alcohol consumption to be a predictor of injury morbidity and mortality, including non-fatal injury in the U.S general population [23] and fatal injury among U.S adults aged above 55 [24].

The association between smoking and injury mortality has previously been observed in literature [15]. This association has been reported to be confounded with alcohol use, however this analysis as well as that of Kawachi et al. (1993) [25] found smoking a significant risk factor for injury death after adjusting for alcohol consumption. Having quit smoking by baseline was not a significant predictor of injury mortality, indicating that personality traits that may cause this association [26] may be changed after quitting smoking. The association between anxiety/depression and injury is often reported in the reverse direction, however the association between anxiety/depression and subsequent injury mortality in the cohort (Table 2) was also seen in a study of adults in a rural American county [14]. Interestingly, we found evidence that this association may be stronger among females. Among males, anxiety/depression was actually significantly associated with non-injury mortality (Table 4), which may be confounded by other serious disease for example. Whilst we found females occasional female drinkers having half the odds of injury mortality compared to never drinkers (Table 3), no literature was found evaluating any of these associations by sex in Thailand. Further analysis is required to understand the cause of this association.

The ongoing political violent conflict in the southern region of Thailand [27] likely explains the elevated risk of injury mortality among southern residents in the cohort (Table 2).

Our study is not without limitations. Data for risk factors was obtained at baseline (2005) whilst deaths included in analysis occurred up until 2016. Lifestyle changes may occur in this time period which effect the interpretation of risk factors. However, it should be reiterated that the research aim was to determine long-term risk factors to injury mortality as opposed to immediate risk-factors. The elderly population was under-represented in our cohort and this should be taken into consideration when reading our findings – the lack of older participants may mean the burden of falls are underrepresented and hence traffic accidents have an abnormally high majority of all injury deaths. Participants were also required to self-report due to the mailed-out questionnaire design of the study, however a large proportion of the cohort reported risk habits such as drink-driving, so this does not appear to be a major issue. There is still a potential for self-report bias in the data reported here. This could potentially mean that the real prevalence of risk behaviours may be underestimated.

Low socio-economic status was also underrepresented in the cohort, which may influence our results. However, one advantage of our study is that all participants provided their Thai Citizen ID number, allowing us to monitor deaths amongst cohort participants into the future. This will allow for ongoing analysis between baseline risk factors and mortality, and whilst our current analysis of injury mortality lacks enough data on elderly participants for meaningful analysis within this group, this analysis will be possible as our cohort ages. Furthermore, logistic regressions for individual causes of injury deaths will be possible in future analysis when the number of cases is high enough for statistically meaningful analysis. Finally, the analyses presented here are assessing risks and associations with injury from all-causes. The ability to conduct cause-specific analyses was hampered by the numbers in each cause category, and the prevalence of non-specific or unknown injury causes (Table 1).

## Conclusions

There were 363 injury deaths in the Thai Cohort Study between 2005 - 2015. We found that historical drink-driving but not alcohol consumption was associated with injury mortality, as well as male sex, smoking, anxiety/depression, and prior injury history. Residing in the southern region of Thailand was a risk factor for injury mortality among males only, whilst the association with anxiety/depression was stronger among females. The association between smoking and alcohol consumption with injury mortality needs to be considered as we found smoking a significant risk factor to injury mortality despite being adjusted for alcohol consumption. The association between residing in southern Thailand and injury mortality may be reflective of political conflict, further research is required to understand this.  

## Data Availability

The datasets used and/or analysed during the current study are available from the corresponding author on reasonable request. The lead author signed a data access agreement for use of the Thai Cohort Study dataset.

## Abbreviations

AOR: Adjusted Odds Ratio
CI: Confidence Interval
ICD: International Classification of Disease
ID: Identification
OR: Odds Ratio
SPSS: Statistical Package for the Social Sciences
STOU: Sukhothai Thammathirat Open University
TCS: Thai Cohort Study
U.S: United States
WHO: World Health Organization
